# Contact tracing: Achieving equilibrium between blockchain solutions and privacy amid the novel coronavirus pandemic

**DOI:** 10.1177/20438869211028869

**Published:** 2021-08-04

**Authors:** Trícia Karla Lacerda Moraes, Adrian Kemmer Cernev, Eduardo Henrique Diniz

**Affiliations:** Fundação Getulio Vargas, Escola de Administração de Empresas de São Paulo (FGV EAESP), Brazil

**Keywords:** Blockchain, GDPR, GDPL, COVID-19, contact tracing, SocialTech, privacy

## Abstract

In moments of scepticism and hopelessness, the drive to satisfy collective needs pushes humanity to accomplish incredible feats to ensure our survival on earth. Indeed, the current global crisis caused by the novel coronavirus is no exception. We are struggling with the abrupt spread of this illness and continuously striving to find efficient solutions. This has been André Salem Alégo’s primary inspiration since the crisis hit his country in March 2020. He is the chief executive officer of Blockforce, a blockchain-based SocialTech. Having observed the effects of novel coronavirus, André examined various approaches that could help contain the spread of the virus and keep society safe. He came up with the idea for an innovative service called Desviralize, which supports pandemic crisis monitoring through contact-tracing with the use of blockchain technology. This teaching case covers how an emergent technology evolves amid the pandemic. It draws attention to important facts related to the digital era such as data security and privacy in the context of Desviralize and Blockforce’s chief executive officer’s strategies for boosting platform growth within the constraints imposed by data protection regulation, the critical pandemic situation, and the ethical aspects of the application of technology.

## Introduction

Who would have anticipated that the entire world would have to quarantine together? Everything changed as of December 2019, when human history experienced a terrifying milestone in the form of the novel coronavirus (COVID-19) crisis. It will certainly change everybody’s life forever, including that of André Salem Alégo, a high-tech entrepreneur who found himself stuck in his apartment in the city of São Paulo on an ordinary Wednesday afternoon in March 2020 due to a government-issued lockdown. He started reflecting on all the aspects of the crisis that has abruptly dictated a new way of living for the global society. While quarantined for 4 weeks, the entrepreneur walked slowly from his home office to his balcony, seeking inspiration to reconcile real-time viral spread monitoring with privacy protection to help humankind contain the crisis. How can technology help him?

André is the founder and chief executive officer (CEO) of Blockforce, a SocialTech start-up that specialises in blockchain solutions aligned with sustainable entrepreneurship aspirations. Considering that COVID-19 is the biggest threat ‘to life or physical safety’ and that the solution depends on the implementation of public policies and rapid, rational, data-based decision making, André focussed on his passion for high technologies, especially blockchain, and dove into intense work, day and night, for the past 3 weeks. He came up with a brilliant idea to help monitor and contain the crisis: Desviralize, a contact-tracing platform aimed at assisting with the epidemiological monitoring of COVID-19. After drafting his new project aims, he decided to share his innovative ideas with some of his partners. He joined two virtual meetings to pitch his project to his lawyers and technical team. In both meetings, he heard suggestions that could substantially affect his new project’s market entry strategies and determine which direction he might take, as well as the likely outcomes.

His lawyers suggested a conservative, cautious approach that rigorously reflects the content of Brazil’s General Data Protection Law (the GDPL, or LGPD in Portuguese); that is, Blockforce’s servers should refrain from storing platform users’ personal information for later use, for instance, to send updated notifications about the pandemic. However, the technical team affirmed that without the notification feature, which encourages users to return to the platform and update their health status, Desviralize would be unable to provide the core service of tracking viral spread and keeping the platform up to date. Such a feature needs to store users’ geolocation (i.e. the person’s geographic position in real time) and phone number without infringing upon privacy rights. How can that be accomplished?

**Figure 1.**
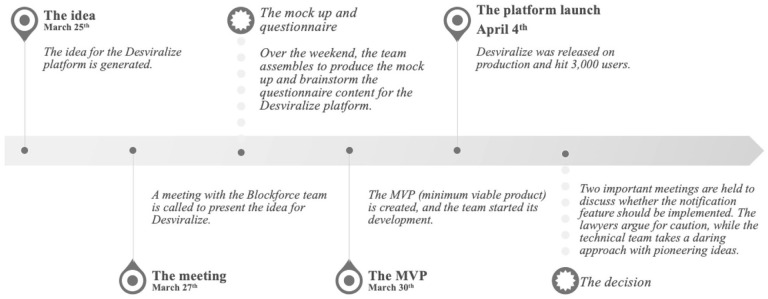
Desviralize timeline – where did the idea come from? *Source*: Own illustration.

Desviralize’s new feature launch date is approaching, and André needs to make a tough decision (see Figure 1). Should he exploit the loopholes in the GDPL, which has not yet been definitively implemented in Brazil, and prioritise the essentiality of users’ geolocation to the notification feature, as supported by his technical team, or should he remove the notification feature to ensure users’ data privacy? Alternatively, he can continue searching for another technical solution to avoid any violation of the GDPL, but this will likely delay the launch. What will he decide?

## COVID-19: the global crisis

Who would have thought that a microscopic virus that is invisible to the human eye could stop the world? Such a thing seemed impossible until COVID-19, the most critical epidemiological crisis in more the one century. COVID-19 is spreading so quickly that people are beginning to realise that the simple countermeasure of staying at home is insufficient. Around the clock, people from countries all over the world are following the news and noticing that in spite of the drastic stratagems imposed by various governments, the number of infections is still rising. Moreover, authorities do not know whether the epidemic has peaked as yet (Sciarra, 2020).

Regarding the high contamination rates due to the circulation of asymptomatic infected people, there is the option of vertical isolation, which entails the confinement of only a few groups of people, namely, those who are the most at risk of developing the disease and suffering from a more severe form of it. Amid the COVID-19 pandemic, the groups that are the most at risk for developing the disease and presenting with a more severe form of it are the elderly and people with pre-existing health problems such as diabetes and cardiovascular diseases. In vertical isolation, only they would be quarantined, while young, healthy people continue to live normally. However, it is unclear whether this measure would be advantageous because young people are significant vectors of the disease, and the number of contaminants could rise rapidly.

In contrast, horizontal isolation does not involve selective quarantine; rather, everyone must remain at home to restrict contact between people as much as possible in an effort to prevent the spread of the disease. Hence, horizontal isolation is widely criticised for its severe negative impacts on the economy, although the approach is often essential to prevent the uncontrolled intensification of the pandemic, which can have consequences like the collapse of the health system and economic loss. Furthermore, it is critical to have up to date and accurate data about deaths and confirmed cases that represent what is happening in a specific scenario in real time in the current situation. However, most countries suffer from underreporting issues due to the lack of sufficient tests to diagnose COVID-19 in patients with symptoms.

This borderless crisis sheds light on the structural deficiencies in those countries that have not worked to diminish inequities; consequently, they will face hard decisions, with minimal resources to care for the most vulnerable. Some national public health systems do not have the necessary infrastructure, and many families will need support because of the cost of medical care and supplies, which could push them deeper into poverty. Many have lost their job because of the crisis. The strain on women is higher, as they are dealing with children who are out of school, as well as grappling with their own employment. Governments have issued decrees to assist small businesses, such as guaranteeing loans, temporary job restructuring, and delayed tax payments; however, realistically, the actual impact on the global economy might be catastrophic.

This prediction is supported by the Coincident Global Barometer reading, which fell 8.5 points in April 2020, from 77.9 points to 69.4 points, hitting its lowest level since May 2009 and thus signalling a deepening slowdown of the global gross domestic product (GDP). Furthermore, the Global Leading Barometer dropped 12.5 points within 1 month, from 86.9% in March to 74.4% in April. Both indicators show that the spread of COVID-19 outside the Asian region has caused a health crisis that is shaking economies on all continents (FGV IBRE News, 2020). Given the economic ravages, nations will continue to deal with the outgrowths even after the COVID-19 crisis has ceased. What should they do?

Recovery is a significant challenge for everyone. The COVID-19 crisis has changed how we look at the world and ourselves. Long periods of confinement have made people realise how vulnerable and interconnected we are. There is an urgent need for universally accessible public services and an efficient tax system to compensate frontline professionals and decision makers who are fighting against the clock, saving lives, and prioritising the basic right to health and wellness.

## Data protection regulation: the dilemma

One of the strategies to combat COVID-19 is to use data collected by mobile phones, including geolocation information. These data can help to accurately estimate the degree of social distancing within a given community. They can also contribute to ensuring that essential activities continue. Countries such as Taiwan, Japan, and South Korea have adopted such strategies with high effectiveness. A question that arises is whether this type of data usage would be allowed under the European Union’s (EU) General Data Protection Regulation (GDPR) models. In Brazil’s context, the equivalent GDPL^1^ predicted situations such as the current crisis (Lemos, 2020). At present, the law authorises the use of data for ‘protecting life or physical safety’, which includes third parties and dispenses with prior consent. The law also allows the use of data for ‘public policy execution’. However, even in emergencies, the usage of user data is restricted, as the duties of observing ‘the general principles and the guarantee of [data subjects’] rights’ remain intact.

According to Spencer Sydow, a digital law specialist and chairman of the Digital Law Commission at the Brazilian Bar Association (OAB, or ‘Ordem dos Advogados do Brasil’ in Portuguese) in São Paulo, regarding corporate responsibility in the context of the GDPL rules, companies must explain how they will use the data they collect from users, disclose the duration for which they will retain the data, and destroy the data after they have served their purpose (Fleischmann, 2020). In other words, even during emergencies, data should only attend specific purposes, and when the emergency ends, the data must be destroyed. The practice of using data without consent has been discontinued. In addition, techniques such as anonymisation and data aggregation should be utilised whenever possible. Finally, the entire process requires transparency and accountability. In Europe, the Data Protection Supervisory Council issued a statement in March 2020, addressed to the European Commission. The critical point in the Brazilian scenario is the August 2020 data protection law, which has been formally enacted and has full force. Its content and regulations already have practical effectiveness and are now followed and applied by companies, public authorities, and the judiciary. Hence, in Brazil, this law should be followed when making decisions about data usage.

## André Salem Alégo: the social entrepreneur

André Salem Alégo is the co-founder and CEO of Blockforce, a São Paulo start-up founded in 2018. Blockforce specialises in blockchain consultancy and digital product development, as well as application-driven social innovation initiatives. André graduated from the Escola de Administração de Empresas de São Paulo at Fundação Getulio Vargas (FGV-EAESP) in 2014 with a bachelor’s degree in business administration and is pursuing a micro masters in data, economics, and policy development at the Massachusetts Institute of Technology (MIT). André began his technology journey in 2006 when he became a professional game player. His nickname was ‘fatan’, and he participated in many Counter-Strike championships with his team ‘semXorah’ (see Appendix A). After finishing high school, he decided to drop game playing as a career and earn his bachelor’s degree in business administration.

His career commenced as a trainee in 2015, and he took advantage of some opportunities before settling at IBM as a blockchain business developer. As a trainee, André had the opportunity to work in various areas, from operations and marketing to being in contact with artificial intelligence (AI). In AI, the benefits and scale of the transformations technology could provide fascinated him, motivating him to research new technologies and their benefits, which led him to blockchain. He researched the challenges and benefits the technology could introduce to society and eventually came to see himself as a blockchain evangelist. This drove him to look for opportunities to work with blockchain at the IBM Research Lab, which boasts a concentration of scientists and studies on new innovative technologies such as AI and blockchain. He had to develop new skills, such as knowledge of software architecture and the Python language. In the Lab, he worked alongside Percival Lucena, a pioneer blockchain specialist in Brazil, who would later partner with him to found Blockforce.

At the IBM Lab, André worked to design blockchain products, reflect market needs in the Lab’s research, and analyse whether the Lab’s studies and efforts matched clients’ expectations. By 2016, given the growth in the area and his experience in customer-centred product design, André moved to the blockchain business unit – a new area created to lead blockchain business in IBM Brazil. With a strong presence among large traditional customers, André saw through this opportunity that the technological potential for innovation was limited. He knew it was possible to decentralise a network, adopt new forms of consensus, and influence the decision-making process; nonetheless, the organisations’ projects in which he was involved did not use blockchain’s full potential, which frustrated him. However, his work triggered new ideas, and André suggested that the Lab could research start-ups that apply blockchain to social projects. Therefore, André worked on the Welight^2^ project through a programme organised in cooperation with the IBM start-up ecosystem. He contributed by applying Hyperledger’s traceability to track activity logs and provide cashbacks that were delivered to a non-governmental organisation (NGO) of the customers’ choice. Welight was an impact initiative that IBM piloted pro-bono.

After the first Welight implementation, IBM had no interest to keep providing support to that company, which raised the opportunity for André to leave IBM and venture into his own blockchain initiative that later becomes Blockforce. During the transition, André contacted an old IBM client, Taynaah Reis, founder of Moeda Seeds,^3^ the first Brazilian project to receive international investments via blockchain. She offered André the opportunity to lead her company’s blockchain development projects, which are now part of Blockforce’s projects. Through this partnership, André stepped into his role as the head of blockchain for Moeda Seeds. Previously, André was the director of innovation and blockchain at Welight. In addition, he acts as a mentor for start-ups in the blockchain segment and has had stints at ConsenSys^4^ for the Blockchain for Social Impact Coalition^5^ (BSIC) and the Blockchain Academy.^6^ He has also participated in blockchain discussion events, such as TEDx Talks.^7^ Appendix B details his experience and achievements.

## Blockforce: the high-tech venture

**Figure 2.**
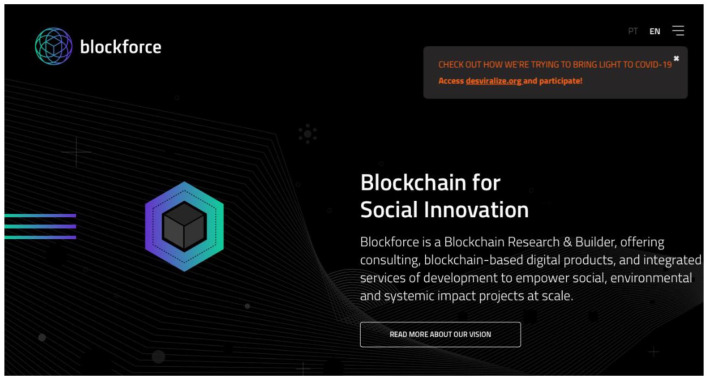
Blockforce website. *Source*: reproduced with permission from Blockforce (2021).

Blockforce is a blockchain-based SocialTech start-up offering consulting, blockchain-based digital products, and integrated development services to empower social, environmental, and systemic impact projects at scale (see Figure 2).

The venture’s purpose is to utilise the power of blockchain to generate new collaborative realities of trust to accelerate positive exponential transformations in various social sectors. The company focuses on consulting and creating trusted networks designed to develop and apply blockchain solutions that incorporate integrated development services with connected and distributed network protocols to add value to a society with greater unity awareness.

Based on their project experience, multi-blockchain protocols, and frameworks, they compress components and structures to offer blockchain development kits and blockchain-based digital products. They are tools aimed at democratising access and speeding up the time it takes to bring impactful solutions to the market. The objective is to facilitate implementation, decrease the cost of solutions, and increase their applicability and effectiveness.

### The Desviralize.org project

#### A solution

The idea for the Desviralize project arose on 25 March 2020 when Blockforce’s CEO André Salem Alégo found himself thinking, while he was quarantined in his apartment, about how to assist communities with recovering from the COVID-19 crisis.

**Figure 3.**
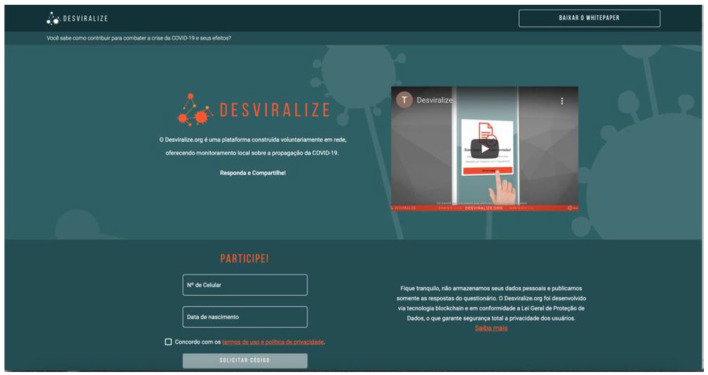
The Desviralize platform. *Source*: reproduced with permission from Desviralize (2021).

The Desviralize platform, which Blockforce developed voluntarily (see Figure 3), is a solution for and by citizens. It proposes to guide epidemiological monitoring based on information from citizens, offering them a general real-time picture of their relationship networks in return. Citizens who sign up will be positioned at the centre of their network. On a map of their residential street, neighbourhood, city, and state, they will then follow the evolution of their direct contacts’ symptoms, within a network of relationships.

Based on the distribution management of citizen-generated data, the platform’s stimulating design offers a simple networked monitoring mechanism to assist with local crisis management. The platform’s objective is to agilely direct treatment and empower citizens’ active role in gathering reliable information and generating socially accountable actions during the pandemic.

The premise of the solution relies on the logic of network sharing and contact tracing, and guarantees the security and authenticity of data from public, immutable, and anonymous registries via blockchain. When accessing the Desviralize platform, users answer a questionnaire about their current health status and share that information with their network of friends, family, neighbours, and colleagues. Users also have access to map visualisations and statistical graphs generated using the data of other respondents in their relationship network (see Appendix C).

The technical team opted for a web system that is accessed via a browser and not a mobile application. André explains that:

With this approach, the platform can be accessed from any mobile device or computer, even for those with less processing power. In addition, location sharing occurs only when the person accesses the system; on the contrary, in a mobile application, the user data is shared all the time, even when the user is not using the application.

#### The challenges

From the viewpoint of citizens consuming news reports about the relentless spread of COVID-19, the first challenge is to access data in real time, since the statistics that reach us via traditional means always illustrate a situation that has already passed, which is not helpful in serious progressive epidemiological scenarios. These statistics represent a picture of ‘yesterday’, as governments obtain them after they have gone through a predetermined hierarchy that includes public and private health systems at the federal, state, and city levels (Lemos, 2020).

The second challenge is to ensure the accuracy of the released data. Many cases are unaccounted for because they do not reach the health systems. This is a sub-notification issue. It occurs because health authorities rightly recommend that people refrain from flooding the health system all at once to avoid generating large crowds of individuals who, although they may have weak symptoms, can be potential disseminators to higher risk communities. However, the disadvantage is that if infected people stay at home, and if some even recover independently, they will not be counted in the statistics, which increases authorities’ lack of awareness regarding the exact number of infected persons.

The third challenge is coverage. Considering all the information the press releases, we are much more in touch with what happens abroad than we are with the local situation in our own neighbourhood. This highlights the need for a solution that helps disseminate helpful local information about the COVID-19 crisis to all citizens, not just the government.

#### The contact-tracing methodology

Contact tracing, commonly known as partner notification, is a convenient method for controlling contagious viruses. As a methodology, it has a long history of use against diseases such as tuberculosis (TB), human immunodeficiency virus (HIV), sexually transmitted diseases (STDs), typhoid, severe acute respiratory syndrome (SARS), and influenza pandemics (Armbruster and Brandeau, 2007). Since the world was notified about the possibility of contracting COVID-19, the meticulous use of contact tracing over digital and physical spheres has been prescribed to limit viral spread in many countries including Singapore, Taiwan, and South Korea, as well as in Kerala and India (Bell, 2020).

The idea of using contract tracing to control viral spread amid the pandemic has raised new concerns about surveillance and privacy. With the launch of a mobile phone app in South Korea to track the virus and monitor citizens during self-quarantine, the trade-offs between health, social well-being, and personal rights have been spotlighted. Indeed, the situation urges us to find a way to balance all these considerations in a successful battle against COVID-19. Countries can negotiate new social contracts that encompass neighbourhoods, communities, and governments to extend the role that technology plays in responding to health crises. As these new contracts are negotiated, enquiries will unavoidably appear concerning our ties to the data that exist around us and the bounty of information that is generated daily, as well as how these can be utilised to serve or harm us (Bell, 2020).

#### The launch

Blockforce’s team developed the Desviralize platform over 2 weeks. On 4 April 2020, the first release reached 3000 users. The project received support from important entities, such as the Google start-up programme,^8^ the Social Technology Institute (ITS Brasil),^9^ and the start-ups Moeda Seeds and Twilio.^10^ To promote the Desviralize platform, Blockforce created a marketing campaign, encouraging users to sign up (see Figure 2) and spread the word via their social networks and websites.

Cointelegraph, a website specialising in blockchain and cryptocurrencies (Gusson, 2020), released the following headline:

Using blockchain, Brazilian start-up Blockforce launches innovative, transparent and 100% free project to map the progress of coronavirus in Brazil.

The above-mentioned headline references Desviralize, a totally free blockchain-based initiative launched in March 2020 with the aim of creating a real-time map to monitor the spread of coronaviruses in Brazil. It is available at the website https://desviralize.org (see Appendix C). Through this initiative, people from all over Brazil can log in and answer a questionnaire indicating whether they have been diagnosed with coronavirus disease and/or are experiencing symptoms, in addition to other questions. Based on the questionnaire responses, the platform categorises users into five categories:

- No symptoms;
- Selected symptoms;
- Fever + 1 symptom;
- In quarantine;
- Recovering.

The map updates in real time and provides a reliable overview of the spread of COVID-19. It also identifies people nearby who may be showing symptoms and/or need special attention. To preserve privacy, users are kept anonymous, and the platform does not share any data on the system. According to Blockforce’s team, Desviralize is:

a blockchain system for monitoring symptoms during the pandemic, developed by a group of close friends. The primary idea is to create a reliable database concerning disease contagion, not only considering people with results tested in hospitals but also with perennial records (When? What symptoms? Had contact with someone infected? Travelled recently? Has health risks? etc.). Respondents are all anonymous, only authenticated by mobile phones, and all the data are open to researchers.

Desviralize platform differentiators are as follows:

- Secure information, unique and anonymous registration of respondents, guarantee of public network history, and immutability via blockchain;
- Dissemination via the network effect, with each user inviting others in their circle of relationships to join the app (viral);
- Anonymisation of respondents;
- Disassociation of identity (phone) and questionnaire responses;
- Local crisis management tooling (companies, condominiums, communities, neighbourhoods);
- It even returns to the crisis through georeferencing;
- Modularisation for a transparent and collaborative census that is adaptable to different situations.

#### The need for a notification feature

Traditional contact monitoring methods limit dissemination. However, technology can extend these efforts using apps to quickly notify people if they have been exposed to someone with COVID-19, including people the infected patient does not know directly. The notification feature in Desviralize will inform users if they have had contact with suspected cases or been contaminated; the feature will show regions with a high probability of contamination and encourage users to return to the platform to update their health status and monitor viral spread.

In this context, notifications can unlock economic activities, in addition to showing the location of the nearest local business, along with user ratings. Making the contagion status available on a public map would support organic and cautious market activity resumption. The same logic can be applied to pharmacies and other essential places where there is a constant flow of people traffic. Regarding hospitals, the users’ report would potentially include a visualisation of estimated bed availability and preparedness for COVID-19, offering a distribution (or at least an indication for public authorities and society at large) of availability and attention areas. Moreover, because Desviralize extends the report’s functionality beyond health symptoms by expanding the scope of the questionnaire, the platform has the potential to empower society, in a registered and immutable way, with regard to the resumption of economic activity and its historical evolution.

### The final decision

The first release of Desviralize grabbed Brazilians’ attention. When questioned about platform technology privacy matters, André responded:

This is a great example of how the use of technology can be neutral and depending on the human behind it we can have ‘Command & Control’, such as Chinese monitoring solutions at the expense of individuals’ privacy, or ‘Connection & Trust’, like Desviralize, which allows anyone to share their location and health data without the other end – government or other citizens – having access to their identity or phone number.

Nevertheless, to sustain growth and exert the platform network effect, André needs to choose his next move, which will determine Desviralize’s future. He will have to carefully assess the pros and cons of implementing the critical notification feature, which might put his business at risk if the platform uses users’ data without anonymisation. However, it will be difficult for him to withdraw his recent affirmation that Desviralize anonymises all its users’ data to guarantee their privacy in favour of contravening the GDPL and exposing users’ data for the ‘greater good’ of helping to battle the pandemic. André is behind the eight ball in terms of choosing between privacy and the crucial notification feature. He has arranged a meeting with his lawyers and technical team to assist him in studying the feasible possibilities.

Having identified all the relevant considerations, André must examine them to make his final choice to address the situation. Adding to the dilemma, he only has a few days to decide and communicate his decision to his stakeholders. He therefore has an important meeting next week with leading investors who are interested in applying the Desviralize blockchain and contract-tracing solutions to other related cases. Moreover, the technical team will have to prioritise the development of Desviralize and implement the chosen solution quickly to facilitate this meeting, and doing so will delay other projects. However, everything built for Desviralize can be used in future projects involving the same technologies.

Now, imagine that you are in André’s shoes. See Appendix D for the description and a timeline of the CEO’s main events. On one hand, you can contribute to battling the pandemic by implementing the notification feature to pioneer a blockchain-based contract-tracing solution that will connect people from all over the country and keep them up to date regarding the spread of COVID-19. On the other hand, there are the GDPL and data privacy matters regarding the usage of users’ data, along with all the implied risks. Indeed, this is a difficult situation. To help the CEO in the decision-making process, it is necessary to identify which option will best suit the Desviralize scenario (see Table 1).

**Table 1.** Options for deciding Desviralize’s future.

Option	Description
(A) Ignore the GDPL	Implement the notification feature and go live without anonymising users’ data
(B) Refrain from implementing the notification feature	Desist with the implementation of the notification feature to comply with the GDPL
(C) Figure out a way to comply with the GDPL	Study how to combine contact tracing with other technologies, such as blockchain, to assure data anonymisation, thus allowing for the implementation of the notification feature, while in compliance with the GDPL
(D) Try to convince regulators that the pandemic situation justifies noncompliance with the GDPL	Even when authorising the use of data in an emergency, the GDPL demands that ‘the general principles and the guarantee of [data subjects’] rights’ remain intact. Therefore, André needs to convince the authorities that the pandemic context justifies adjusting the data protection law to allow for exceptions
(E) Hand the Desviralize platform over to the government	Hand the Desviralize platform over to the government, so that they can continue with the project and implement the notification feature

*Source*: Own table.

GDPL: Brazil’s General Data Protection Law.

After carefully analysing the information you have in hand, it is time to decide which action you would take and why.

## Figures and Tables

**Figure A1.**
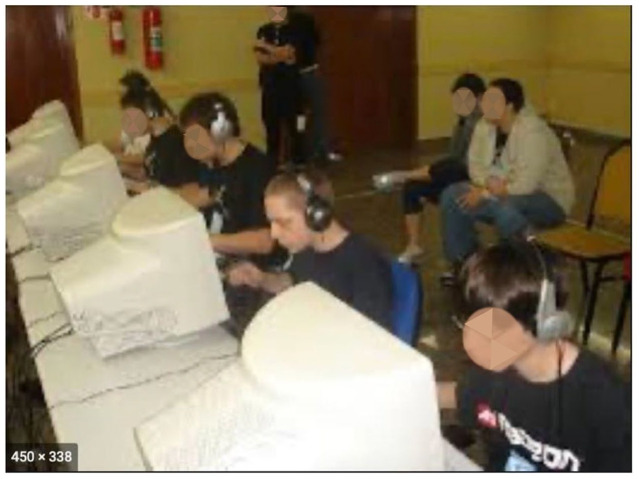
André ‘fatan’ Salem – the game player. *Source*: André’s personal archive (he is the second player, from the right corner to the left).

**Figure C1.**
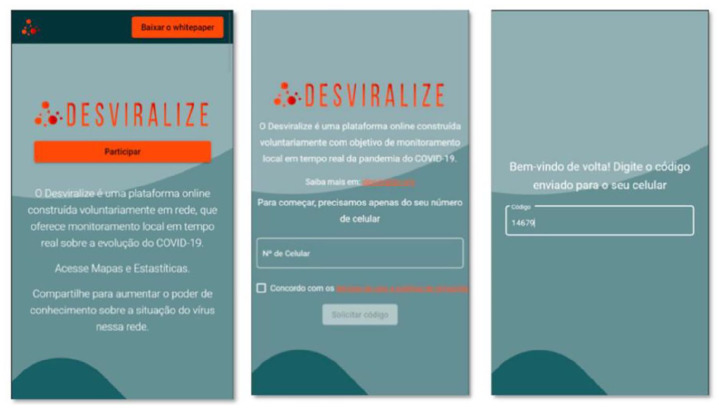
Desviralize platform – log in screens. *Source*: Desviralize (2021).

**Figure C2.**
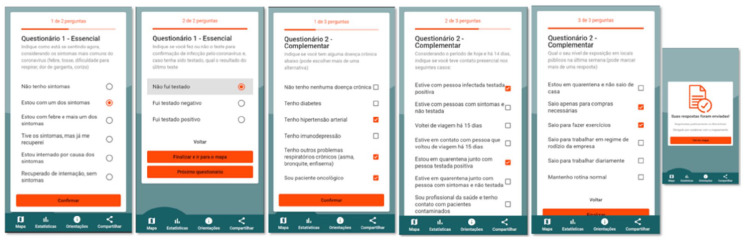
Desviralize platform – questionnaire. *Source*: Desviralize (2021).

**Figure C3.**
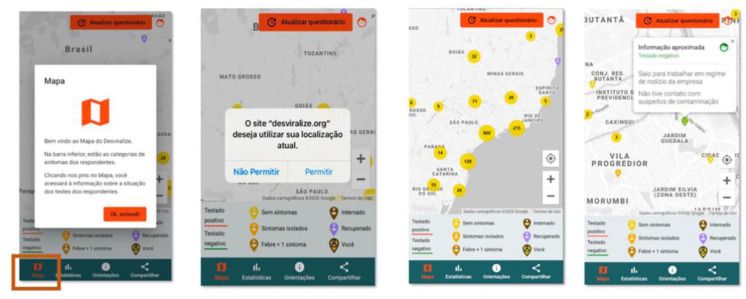
Desviralize platform – maps. *Source*: Desviralize (2021).

**Figure C4.**
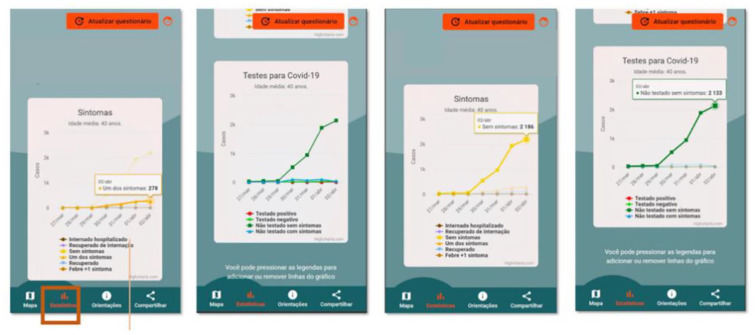
Desviralize platform – statistics. *Source*: Desviralize (2021).

**Figure C5.**
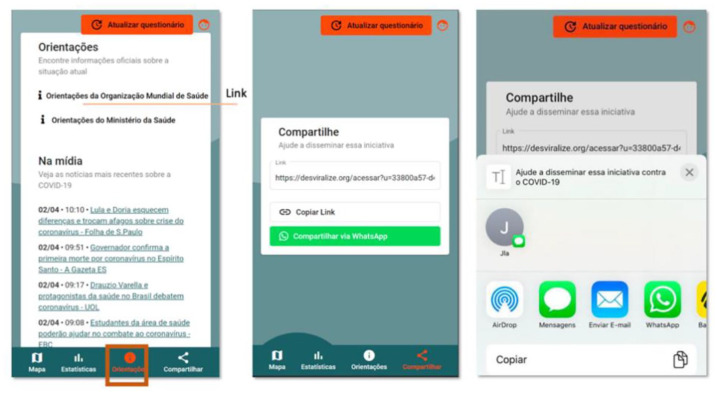
Desviralize platform – orientation. *Source*: Desviralize (2021).

## Appendices

### Appendix B

**Table B1.** André Salem Alégo and Blockforce – experiences, achievements, and publications.

Year	Month	Description	Media	Source
2020	Apr.	Shawee: Hack for Good: André Salem and Elisa Guimarães Cardamone presenting impact cases using Blockchain (in Portuguese; automatically generated English subtitles available)	Video	https://www.youtube.com/watch?v=80Ol2yufyfw
2019	Oct.	BlockForce: Chamada Din4mo-FIIMP 2 – YouTube (in Portuguese; automatically generated English subtitles available)	News	https://www.youtube.com/watch?v=8l5qZsJDrwI
2019	Sep.	Voicers: Blockchain para migrarmos da Ganância para Confiança–Tecnomagia #08 (in Portuguese; automatically generated English subtitles available)	Video	https://www.youtube.com/watch?v=EQ6icoFEz3c
2018	Nov.	FGVces–FGV EAESP: Drops – Descomplicando o Blockchain (in Portuguese; automatically generated English subtitles available)	Video	https://www.youtube.com/watch?v=_QV3tewlQNA
2018	Nov.	What is blockchain and how it is applied to certification? (in English)	News	https://www.p22on.com.br/en/2018/11/02/o-que-e-blockchain-e-como-se-aplica-certificacao/
2018	Sep.	Blockchain Academy oferece curso exclusivo sobre Hyperledger (in Portuguese)	Course	https://blockchainacademy.com.br/blockchain-academy-oferece-curso-exclusivo-sobre-hyperledger/
2018	Sep.	TEDx Talks: Blockchain e impacto social – André Salem – TEDxMauá (in Portuguese; automatically generated English subtitles available)	Video	https://www.youtube.com/watch?v=LqLA-PGAb-o
2018	Jul.	Moeda.TV: People – Head of Blockchain (in Portuguese; automatically generated English subtitles available)	Video	https://www.youtube.com/watch?v=yZamAfmUa6s
2018	Apr.	Blockchain Insper: Blockchain nas instituições governamentais (in Portuguese)	News	https://medium.com/blockchain-insper/blockchain-nas-institui%C3%A7%C3%B5es-governamentais-43996bc0be7e
2018	Mar.	Cotidiano Aceleradora: Blockchain vai transformar o mundo – com André Salem (in Portuguese; automatically generated English subtitles available)	Video	https://www.youtube.com/watch?v=zU8fTjLvIEo
2017	Oct.	Conferência Web.br 2017 Workshop titled ‘Blockchain para muito além de criptomoedas – parte 2’ (in Portuguese; automatically generated English subtitles available)	Video	https://www.youtube.com/watch?v=_oWTQXkBdy4
2017	Oct.	Conferência Web.br 2017 Workshop titled ‘Blockchain para muito além de criptomoedas – parte 1’ (in Portuguese, automatically generated English subtitles available)	Video	https://www.youtube.com/watch?v=fk_VQzQinLY
2017	Oct.	Conferência Web.br 2017 Blockchain Beyond Finance (in Portuguese)	Presentation	https://ceweb.br/webbr2017/apresentacoes/1-Andre%CC%81%20Salem-Blockchain_VF.pdf
NS	NS	André Salem – LinkedIn profile (in English)	News	https://br.linkedin.com/in/andresalem
NS	NS	Crunchbase – André Salem	News	https://www.crunchbase.com/person/andré-salem
NS	NS	Crunchbase – Blockforce (in English)	News	https://www.crunchbase.com/organization/blockforce#section-overview
				

*Source*: Own table.

NS: not specified.

### Appendix D

**Table D1.** Desviralize and its CEO’s main events timeline.

Year	Month	Event description
2020	Apr.	Desviralize platform’s first release goes live (without the notification feature)
2020	Mar.	The Desviralize MVP (Minimum Viable Product) is created
2020	Mar.	Meeting with the Blockforce team to present the Desviralize project idea on 27 March 2020
2020	Mar.	The idea for the Desviralize project into being on 25 March 2020

*Source*: Own table.

CEO: chief executive officer; NS: not specified.

## Notes

1. https://iapp.org/resources/article/brazilian-data-protection-law-lgpd-english-translation/.

2. https://welight.co.

3. https://moedaseeds.com.

4. https://consensys.net/.

5. https://blockchainforsocialimpact.com/.

6. https://blockchainacademy.com.br/?lang=en.

7. https://www.ted.com/talks/andre_salem_blockchain_e_impacto_social.

8. https://startup.google.com.br/.

9. https://itsbrasil.org.br.

10. https://www.twilio.com.
